# A guide for the management of post vaccination allergy and anaphylaxis in a pharmacy clinic

**DOI:** 10.4102/hsag.v27i0.1987

**Published:** 2022-11-15

**Authors:** Ané Orchard, Muhammed Vally, Razeeya Khan, Mohamed Irhuma

**Affiliations:** 1 Department of Pharmacy and Pharmacology, Faculty of Health Sciences, University of the Witwatersrand, Johannesburg, South Africa

**Keywords:** emergency tray, injection, algorithm, allergy, anaphylaxis, adrenaline

## Abstract

**Background:**

Vaccination falls within the scope of practice of a pharmacist and the coronavirus disease 2019 pandemic has seen an increase in pharmacies providing vaccination services. These vaccines are not without risk of allergic reactions and anaphylaxis. The available guidelines for the management of anaphylaxis include the administration of intravenous (IV) fluids. However, IV administration does not fall within the scope of practice of a pharmacist. A gap was identified in the availability of guidelines for the management of anaphylaxis without the use of IV fluid administration.

**Aim:**

This review aimed to address this gap by describing the mechanisms of allergic reactions and anaphylaxis and developing an algorithm to assist pharmacy personnel to manage these within the scope of practice.

**Methods:**

The authors used the recommendations for developing guidelines.

**Results:**

The availability of anaphylaxis guidelines and clinical studies catering for anaphylaxis and allergy management by pharmacists was deficient, thus the review modified the available management guidelines to align the management of allergy and anaphylaxis within the scope of a pharmacist. The items required for the management were also identified and listed as items that form part of the emergency tray in the pharmacy.

**Conclusion:**

The review designed algorithms based on the available literature to assist pharmacy personnel to manage allergy and anaphylaxis within the relevant scope of practice. The review also lists the equipment needed for an emergency tray.

**Contribution:**

This review serves to offer guidance for the management of allergy and anaphylaxis in a pharmacy setting.

## Introduction

On 16 August 2021, Business for South Africa (B4SA) listed at least 790 private sector businesses, including both independent community pharmacies and corporate pharmacy chains, accepting eligible persons for the coronavirus disease 2019 (COVID-19) vaccine (Business for SA 2021). The pharmacy profession has joined the vaccination of the community on a large scale.

Human resource strengthening is integral to the introduction of new vaccines (Baleta, Van den Heever & Burnett 2012). Pharmacists are recognised for their role in the storage, preparation and distribution of vaccines; however, their role in the administration of immunisations in the public sector has not been advanced (Reddy et al. 2021). As pharmacists are the most accessible healthcare providers in the community, their role in immunisation services in the private and public sectors should be harnessed to enhance service delivery. The Good Pharmacy Practice Guidelines (GPPs) (South African Pharmacy Council [SAPC] 2021) advises that immunisations must be performed by a pharmacist who has adequate training, knowledge and skills to provide the service. This includes the appropriate qualifications to handle any unfortunate serious adverse events following immunisation. The onus is thus on the pharmacist to be equipped with such required training and skills, as outlined in the recently accredited SAPC course on Immunisation and Injection Technique for pharmacists. Pharmacies intending to offer the services should comply with the minimum requirements for such a service as outlined in Section 2.14 in the GPP (SAPC 2021). These include minimum requirements for the physical facilities and equipment (Sect. 2.14.1), the immunisation procedure (Sect. 2.14.2), documentation and reporting on immunisation activities (Sect. 2.14.3) and the ethical aspects relating to immunisation (Sect. 2.14.4).

Some university programmes for pharmacy undergraduates in South Africa incorporate immunisation skills training as part of the curriculum. As such, these students have the necessary skills to administer immunisations once they graduate. These training programmes, however, are not standardised, nor validated across the universities incorporating immunisation as part of the undergraduate curriculum. In the light of the COVID-19 pandemic, and the roll out of vaccines across the country for target populations, it is imperative that pharmacists join the immunisation task force to assist the government in attaining immunisation goals (Perumal-Pillay 2021). Trained student volunteers would likely improve coverage in mass immunisation campaigns where immediate access to emergency medical service practitioners is available to manage any serious adverse events following immunisation (AEFI). Anaphylaxis is extremely rare yet potentially life-threatening and should be anticipated in any vaccine recipient (Lakshman & Finn 2000).

Although the occurrence of anaphylaxis following immunisation is extremely low, 2.5–11.1 per million for the COVID-19 vaccine (Blumenthal et al. 2021) and 1–1.31 per million for the flu vaccine (McNeil et al. 2016), the large numbers of vaccinations being administered may result in an increased exposure to AEFI. Vaccines are not the only risk to anaphylaxis, there is also a risk from food, insect sting and medication (Terrie 2009). In rare cases, anaphylaxis can be idiopathic, when no specific trigger can be identified after an appropriate evaluation (Fenny & Grammer 2015).

Epinephrine (adrenaline), a non-selective adrenoreceptor agonist, is critical for the management of anaphylaxis (Terrie 2009; Campbell & Kelso 2021). Pharmacists are rarely well equipped to properly deal with anaphylaxis unless they are familiar with the epinephrine autoinjector (Salter et al. 2014). An emergency medical service also needs to be called as soon as possible once it has been identified that a patient is experiencing anaphylaxis; however, the experienced delay in emergency medical service response time in South Africa (Mkize 2016; Finlayson 2017) places patients at an increased risk, especially considering that anaphylaxis can lead to severe morbidities or even mortality within minutes if not managed appropriately and timeously (Campbell & Kelso 2021).

Currently, available protocols and guidelines related to acute allergy and anaphylaxis recommend the administration of specific (case-based) therapies, such as oxygen, antihistamine, epinephrine (adrenaline), glucocorticosteroids, resuscitation fluids (e.g. Ringers lactate), antacids (e.g. ranitidine/cimetidine), glucagon and bronchodilators (e.g. salbutamol/ipratropium bromide) (Allergy Society of South Africa 2021; The Resuscitation Council of Southern Africa 2021a, 2021b). The route of administration includes the intramuscular (IM), inhaled and intravenous (IV) routes. Oral therapy administration is seldom used in emergency presentations. This highlights that current anaphylaxis management guidelines include IV administration, which is not within the scope of practice of a pharmacist.

The GPP (SAPC 2021) outlines the minimum standards to provide an immunisation service. These include the physical facilities and equipment required for the management of anaphylaxis. The current guidelines for pharmacists to manage AEFI are insufficient. In fact, guidelines on allergy and anaphylaxis management tailored to the scope of practice of a pharmacist is lacking, which may lead to confusion regarding the IV infusion, the need to immediately call for an ambulance, indicating that the priorities that a pharmacist would need to focus on may differ. With increased vaccination services being offered in pharmacies, it will be increasingly important for pharmacists to review AEFI management. This review aims to provide the pharmacy community with guidelines on the management of AEFI with algorithmic steps that fall within the scope of practice of a pharmacist, while also providing a list of equipment that should be available on the emergency tray of the practice site.

## Methods

Recommendations as stipulated by Shekelle et al. (1999) were used for developing guidelines. The steps included (1) the defining and refining of the subject area, (2) the identification, synthesis and interpretation of the relevant evidence to produce the resulting guideline, (3) the translation of evidence into the recommendation within a clinical practice setting (which is a pharmacy clinic) and (4) that the guideline was reviewed by a clinician to ensure clinical veracity.

The defining of the subject area involved a broad search of the various definitions and classifications of allergy and anaphylaxis. The subject areas included defining allergy and anaphylaxis and describing the difference between the two. The emergency tray was the next subject area to be addressed as it is not clear in the GPP what items are required in a pharmacy. Thus, the list of items could be identified based on reviewing the available literature on anaphylaxis guidelines. The final subject area was the management of allergy and anaphylaxis in a pharmacy in a manner that only includes aspects relevant to the pharmacist as the current guidelines and algorithms were not aimed at what is available and within the scope of a pharmacist.

The main difficulty with this review is that available anaphylaxis guidelines and clinical studies cater for health professionals for whom IV administration is included in their training. Pharmacists are not trained to administer IV infusions, and this guidance serves to assist the pharmacist to manage AEFI’s within their scope of practice.

### Ethical considerations

An ethical waiver for this research was obtained from the Human Research Ethics Committee (Medical) of the University of the Witwatersrand (reference number R14/49), South Africa.

## Review findings

### Signs and symptoms

#### Allergy

Allergic reactions may present with any of the following symptoms: urticaria/hives (although 10% – 20% of patients have no skin presentations), angioedema, rhinoconjunctivitis, bronchospasm and/or wheezing (Kroger, Bahta & Hunter 2021).

#### Anaphylaxis

This presents as approximately 40 different signs and symptoms in various combinations (Joyce & Lin 2018; Motosue et al. 2017; Sampson et al. 2006). Box 1 illustrates the common signs and symptoms of anaphylaxis (Campbell et al. 2014; Cardona et al. 2020; Ewan et al. 2010; Oswalt & Kemp 2007; Shaker et al. 2020; Simons 2006, 2010).

BOX 1 Common signs and symptoms of anaphylaxis.

The common signs and symptoms of anaphylaxis are as follows:

- Skin and mucosal – hives across the body, itching, reddening of the skin, oedema of the lips-tongue-uvula, oedema of the periorbital area or conjunctival swelling.
- Respiratory – nasal discharge and congestion, sneezing, itching of the throat or ear canals, voice change, throat closure or choking sensation, stridor, dyspnoea, wheezing and cough.
- Gastrointestinal – nausea, vomiting, diarrhoea and crampy abdominal pain.
- Cardiovascular and neurological (because of drop in blood pressure) – tachycardia, hypotension, hypotonia (decreased muscle tone), syncope (loss of consciousness), incontinence and dizziness/vertigo.

*Source:* Campbell, R.L., Li, J.T., Nicklas, R.A. & Sadosty, A.T., 2014, ‘Emergency department diagnosis and treatment of anaphylaxis: A practice parameter’, *Annals of Allergy, Asthma & Immunology* 113, 599–608. https://doi.org/10.1016/j.anai.2014.10.007; Cardona, V., Ansotegui, I.J., Ebisawa, M., El-Gamal, Y., Rivas, M.F., Fineman, S. et al., 2020, ‘World allergy organization anaphylaxis guidance 2020’, *World Allergy Organization Journal* 13(10), 100472. https://doi.org/10.1016/j.waojou.2020.100472; Ewan, P., Dugué, P., Mirakian, R., Dixon, T., Harper, J. & Nasser, S., 2010, ‘BSACI guidelines for the investigation of suspected anaphylaxis during general anaesthesia’, *Clinical & Experimental Allergy* 40, 15–31. https://doi.org/10.1111/j.1365-2222.2009.03404.x; Oswalt, M.L. & Kemp, S.F., 2007, ‘Anaphylaxis: Office management and prevention’, *Immunology and Allergy Clinics of North America* 27(2), 177–191. https://doi.org/10.1016/j.iac.2007.03.004; Shaker, M.S., Wallace, D.V., Golden, D.B., Oppenheimer, J., Bernstein, J.A., Campbell, R.L. et al., 2020, ‘Anaphylaxis – A 2020 practice parameter update, systematic review, and Grading of Recommendations, Assessment, Development and Evaluation (GRADE) analysis’, *Journal of Allergy and Clinical Immunology* 145, 1082–1123. https://doi.org/10.1016/j.jaci.2020.01.017; Simons, F.E.R., 2006, ‘Anaphylaxis, killer allergy: Long-term management in the community’, *Journal of Allergy and Clinical Immunology* 117, 367–377. https://doi.org/10.1016/j.jaci.2005.12.002; Simons, F.E.R., 2010, ‘Anaphylaxis’, *Journal of Allergy and Clinical Immunology* 125, S161–181. https://doi.org/10.1016/j.jaci.2009.12.981

In general, skin and mucosal signs and symptoms occur in 90% of episodes, while respiratory signs and symptoms occur in 85% of episodes (Campbell et al. 2015). Both gastrointestinal and cardiovascular-related signs and symptoms occur in approximately 45% of all episodes (Campbell et al. 2014; Cardona et al. 2020; Ewan et al. 2010; Oswalt & Kemp 2007; Shaker et al. 2020; Simons 2006, 2010). The upper or lower airway obstruction from cardiovascular collapse results in asphyxiation (oxygen deprivation), which results in death (Bock, Muñoz-Furlong & Sampson 2001; Pumphrey 2000, 2004).

Anaphylaxis usually occurs within seconds to minutes of exposure; however, in rare cases it may occur hours later (Simons 2006). It tends to have a rapid onset, evolution and ultimate resolution of signs and symptoms. Because of the poorly understood erratic course of anaphylaxis, neither severity nor the resolution of anaphylaxis can be predicted. Even if one can ‘stabilise’ anaphylaxis, biphasic or protracted reactions may still occur. Regardless, however, of the course of anaphylaxis, the most essential medicine for preventing death is the initial and immediate administration of epinephrine (Simons 2006).

### Biphasic versus protracted versus delayed anaphylaxis

Successful management of anaphylaxis may lead to a seeming resolution of the initial anaphylactic episode; however, a recurrence of the reaction may occur despite there being no further exposure to the causative agent, this is called biphasic anaphylaxis (Alqurashi et al. 2015; Grunau et al. 2014; Lee et al. 2015). The rate of occurrence of a biphasic reaction can range between 0.4% and 14.7% (depending on the population group under study i.e. adult’s vs. paediatrics) and is estimated to occur in approximately 5% of cases overall. Biphasic anaphylaxis usually occurs within 12 h after the resolution of the initial signs and symptoms but can occur as late as 72 h following the initial episode (Alqurashi et al. 2015; Grunau et al. 2014; Lee et al. 2015).

An anaphylactic reaction lasting hours, days or in extreme cases weeks is called protracted anaphylaxis (Sampson, Mendelson & Rosen 1992). Where anaphylaxis often occurs within minutes, for delayed anaphylaxis, the reaction occurs several hours after the exposure (Commins et al. 2016).

### Anaphylaxis versus anaphylactoid

Both anaphylaxis and anaphylactoid events are life-threatening reactions. Anaphylaxis is because of an immediate hypersensitive systemic reaction caused by a rapid IgE-mediated immune release of potent mediators from tissue mast cells and peripheral blood basophils, while an anaphylactoid reaction is triggered by non-IgE-mediated events resulting in the release of mast cells and basophils. The anaphylactoid reactions are predominantly immediate, however, may be delayed, depending on the exposure route, and is generally dependent on systemic exposure to an offending agent in amounts greater than would be expected to elicit anaphylaxis. The occurrence of these reactions is usually immediate, however, may be delayed and occasionally be biphasic (Douglas et al. 1994).

### Diagnosis

#### Allergy

Mild allergic reactions may appear as urticaria and/or redness or itching on the face and skin. It may include swelling of face, eyes, hands and feet, and may progress to stomach pain, vomiting and/or diarrhoea (Cincinnati Children’s, n.d.; Allergy Society of South Africa 2021). Patients may experience chest tightness, wheezing, shortness of breath and/or coughing. A mild allergic reaction generally does not affect the cardiovascular system; however, allergic reactions can progress into anaphylaxis (Kroger et al. 2021).

#### Anaphylaxis

There are essentially three criteria for anaphylaxis. The diagnostic criteria, which include the full spectrum of signs and symptoms of anaphylaxis, are summarised in Table 1 (Sampson et al. 2006).

**TABLE 1:** A summary of diagnostic criteria for anaphylaxis.

Criteria	Explanation
1	Acute onset (minutes to several hours) of signs and symptoms involving the skin, mucosal tissue or both (e.g. generalised hives, pruritis or flushing, swollen lips, tongue uvula) with at least ONE of the following: Respiratory compromise (e.g. dyspnoea, wheeze or bronchospasm, stridor, reduced peak expiratory flow rate, hypoxemia) OR Reduced Blood pressure (BP) or associated symptoms and signs of decreased perfusion to end-organs (e.g. hypotonia, syncope and incontinence)
2	Two or more of the following that occur rapidly after exposure to a likely allergen for that patient (minutes to several hours) Involvement of the skin-mucosal tissue (e.g. generalised hives, itch-flush and swollen lips-tongue-uvula) Respiratory compromise (e.g. dyspnoea, wheeze/bronchospasm, stridor, reduced peak expiratory flow and hypoxemia) Reduced BP or associated symptoms and signs of decreased perfusion to end-organs (e.g. hypotonia, syncope and incontinence) Persistent gastrointestinal symptoms and signs (e.g. crampy abdominal pain and vomiting) The second criteria incorporate gastrointestinal symptoms in addition to the skin, respiratory and BP symptoms
3	Reduced BP after exposure to a known allergen for that patient (minutes to several hours): Reduced BP in adults is defined as a systolic BP of less than 90 mmHg or greater than 30% decrease from that person’s baseline In infants and children, reduced BP is defined as low systolic BP (age specific)* or greater than 30% decrease in systolic BP

*Source*: Taken from Sampson, H.A., Muñoz-Furlong, A., Campbell, R.L., Adkinson Jr, N.F., Bock, S.A., Branum, A. et al., 2006, ‘Second symposium on the definition and management of anaphylaxis: Summary report – Second National Institute of Allergy and Infectious Disease/Food Allergy and Anaphylaxis Network symposium’, *Journal of Allergy and Clinical Immunology* 117(2), 391–397. https://doi.org/10.1056/NEJM199208063270603

* , Key: Low systolic BP for children is defined as: less than 70 mmHg for 1 month up to 1 year; less than (70 mmHg + [2 × age]) from 1 to 10 years, less than 90 mmHg from 11 to 17 years.

### Differential diagnosis

Certain conditions presenting with similar signs and symptoms to anaphylaxis can occur in both adults and children. Common conditions that mimic anaphylaxis reactions include generalised urticaria, acute angioedema, acute severe exacerbations of asthma, syncope as well as panic attacks or acute anxiety (Cardona et al. 2020; Dreskin & Stitt 2020; Sampson et al. 2006). The differences between these conditions and anaphylaxis are illustrated in Table 2 (Cardona et al. 2020; Dreskin & Stitt 2020; Sampson et al. 2006).

**TABLE 2:** Common conditions that should be considered in the scenario of anaphylaxis.

Acute generalised urticaria and/angioedema	Acute severe exacerbation of asthma	Vasovagal syncope	Panic attack/acute anxiety
These symptoms may be associated with anaphylaxis or may occur as an isolated problem Urticaria with or without angioedema is generally limited to the skin and subcutaneous tissue, while anaphylaxis will involve other organs as described here Urticaria can be associated with exposure to an allergen, as well as infections or physical stimuli such as heat or cold There are both allergic and nonallergic causes associated with angioedema	A sudden onset of wheeze, cough and shortness of breath may be because of anaphylaxis but also may occur because of acute severe asthma The diagnosis of anaphylaxis should be considered when patients prevent with respiratory, Gastrointestinal tract (GIT) and cardiovascular symptoms Furthermore, the diagnosis of anaphylaxis should be considered in patients who present with respiratory symptoms within minutes to a few hours after exposure to a likely or known allergic trigger, e.g. a medicine or vaccine	Syncope may be a symptom of anaphylaxis or may occur because of an isolated problem Vasovagal syncope is generally associated with pallor, diaphoresis, weakness, nausea and bradycardia Vasovagal syncope is relieved by a recumbent position Anaphylaxis tends to be more sudden in onset and is characterised by flushing rather than pallor as well as a sudden onset of itching, urticaria, angioedema, hoarseness, throat tightness, and other symptoms as previously described Patients with anaphylaxis are more likely to have tachycardia than bradycardia	In a panic attack or acute anxiety, symptoms include a sense of impending doom, breathlessness, flushing, sweating, trembling, palpitations, globus sensation, light-headedness, chest pain and numbness or tingling of the extremities Some of these signs and symptoms may occur in anaphylaxis, but anaphylaxis also presents with itching, urticaria, angioedema, hoarseness, stridor, wheezing, coughing, hypotension and collapse

*Source*: Cardona, V., Ansotegui, I.J., Ebisawa, M., El-Gamal, Y., Rivas, M.F., Fineman, S. et al., 2020, ‘World allergy organization anaphylaxis guidance 2020’, *World Allergy Organization Journal* 13(10), 100472. https://doi.org/10.1016/j.waojou.2020.100472; Dreskin, S. & Stitt, J., 2020, ‘Anaphylaxis’, in A.W. Burks, S.T. Holgate, R.E. O’Hehir, D.H. Broide & L.B. Bacharier (eds.), *Middleton’s allergy: Principles and practice*, p. 1228, Elsevier, Philadelphia, PA; Sampson, H.A., Muñoz-Furlong, A., Campbell, R.L., Adkinson Jr, N.F., Bock, S.A., Branum, A. et al., 2006, ‘Second symposium on the definition and management of anaphylaxis: Summary report – Second National Institute of Allergy and Infectious Disease/Food Allergy and Anaphylaxis Network symposium’, *Journal of Allergy and Clinical Immunology* 117(2), 391–397. https://doi.org/10.1056/NEJM199208063270603

Other respiratory events that may be a differential diagnosis for anaphylaxis include vocal cord dysfunction, choking/foreign body aspiration, epiglottitis, pulmonary embolism, pneumothorax and/or hyperventilation (Cardona et al. 2020). Other cardiac events that may be a differential diagnosis for anaphylaxis include sudden collapse without skin symptoms or signs, hence the possibility for diagnostic confusion (Cardona et al. 2020). The differential diagnosis for collapse or syncope is broad and includes cardiac aetiologies including myocardial ischaemia, cardiac arrhythmias and/or structural cardiac disease (Cardona et al. 2020). It is worth noting that elevated tryptase levels can be found in some patients with a myocardial infarction and anaphylaxis (Cardona et al. 2020).

### Emergency tray

As is stipulated in the GPP (SAPC 2021), an emergency tray needs to be available in the pharmacy. Box 2 lists the recommended emergency medicines, equipment and consumables for the management of allergic reactions and anaphylaxis as recommended in the Standard Treatment Guidelines and Essential Medicines List for South Africa Primary (The National Department of Health 2020).

BOX 2 Emergency medicines and equipment for the management of anaphylaxis in a pharmacy.

**Table:** 

Guideline and protocol sheet (laminated booklet)
Epinephrine (adrenaline) 1 mg/mL (1:1000) 1 mL ampoule (or adrenaline auto-injector/junior auto-injector – note the very short shelf life)
Hydrocortisone (100 mg/mL 200 mg/2 mL vial)
Nebuliser
Salbutamol 0.5% 20 mL nebulising solution OR 2.5 mg/2.5 mL OR 5 mg/2.5 mL unit dose vial for nebulisation OR salbutamol 100 mcg metered dose inhaler
Ipratropium bromide 0.25 mg/2 mL OR 0.5 mg/2 mL unit dose vial for nebulisation
Promethazine hydrochloride 25 mg/2 mL ampoule
Disposable syringes in appropriate graduations
Needles for intramuscular injections (26 G, and other required difference sizes)
Tourniquet
Consumables (alcohol swabs, adhesive tape and cotton pad)
Sharps container
†Normal saline 0.9% infusion
†IV administration set
‡Automated external defibrillator (AED)

*Source*: Adapted from Board Notice 241 of 2021 of the National Department of Health, 2020, *South Africa: Essential drugs programme. Primary healthcare standard treatment guideline and essential medicine list*, South African National Department of Health, Pretoria.

† , Contents of an anaphylaxis emergency tray include intravenous 0.9% NaCl, the administration of which is outside the scope of practice of a pharmacist. In pharmacies where personnel trained in inserting an IV line are employed, these items would be included in the emergency tray.

‡ , An AED could be added to the emergency equipment in pharmacies where personnel trained in the administration of advanced cardiopulmonary resuscitation are employed.

Currently, a pharmacist with an Immunisation and Injection Technique certification and a Section 22A(15) permit issued by the Director General of Health is permitted to manage anaphylaxis. According to the Bill of Rights, chapter 2 of the Constitution of the Republic of South Africa, section 11, ‘Everyone has the right to life’, which makes it imperative for all community pharmacists to be trained in managing anaphylaxis.

There should be easy access to the emergency medicines required for the management of anaphylaxis. The contents of the emergency tray should routinely be verified as part of the standard operational procedures for expiry dates, integrity of packaging and consumables. Other equipment should be validated as per the recommendation from the manufacturer. A recommendation is to carry out drills in the workplace to ensure that immediate action is possible in case of an emergency because of anaphylaxis. Up-to-date emergency contact details of ambulance services and nearby referral facilities should be available. Valid certification for the provision of first aid, basic life support (BLS) and advanced cardiac life support (ACLS) should be maintained and renewed.

### Treatment

#### Allergy

**FIGURE 1:**
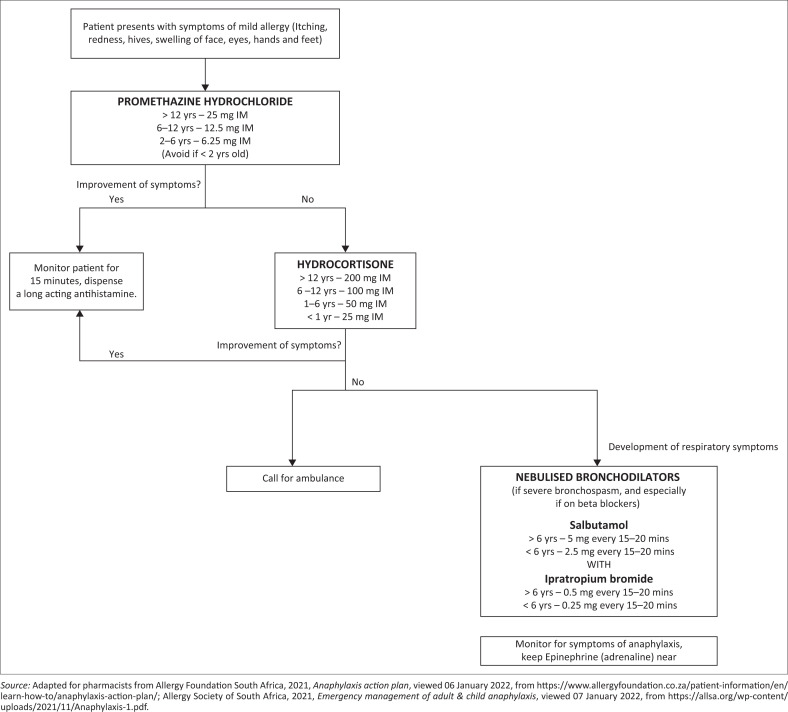
Allergy algorithm.

An allergy generally requires the administration of an antihistamine such as promethazine hydrochloride. If the patient is asthmatic, salbutamol should be administered 6–10 puffs via spacer (Allergy Society of South Africa 2021). The dosages for promethazine hydrochloride, from Allergy Society of South Africa (2021) are given in the allergy algorithm (Figure 1). If the allergy progresses, with no cardiovascular or respiratory symptoms, hydrocortisone (slow IV/IM) or any pharmacologically equivalent glucocorticosteroids should be added. Nebulised bronchodilators should be added if respiratory symptoms occur.

It is recommended to keep epinephrine (adrenaline) close at hand in case of progression of symptoms and the patient should be monitored closely for signs of severe reaction, for at least 15 min (Allergy Foundation South Africa 2021; Allergy Society of South Africa 2021; Kroger et al. 2021). Long-acting oral antihistamines (when possible) should be prescribed after the initial symptoms have been managed (The National Department of Health 2020).

#### Anaphylaxis

Prompt and immediate response to anaphylaxis is required (Bock et al. 2001; Pumphrey 2000, 2004; Sampson et al. 1992). This is because respiratory arrest or cardiac arrest or death can occur within minutes (Bock et al. 2001; Pumphrey 2000, 2004; Sampson et al. 1992). A review of 164 fatalities because of anaphylaxis reported the median time interval between the onset of symptoms and either respiratory arrest or cardiac arrest to be 5 min in iatrogenic anaphylaxis, 15 min in stinging insect venom-induced anaphylaxis and 30 min in food-induced anaphylaxis (Pumphrey 2000). It is of vital importance to treat anaphylaxis promptly because treatment is most responsive in the early phases (Bock et al. 2001; Pumphrey 2000, 2004), and this is based on the observations that delayed epinephrine injection is associated with a greater risk of fatalities (Bock et al. 2001, Pumphrey 2000, 2004). There is no absolute contraindication to using epinephrine in anaphylaxis (Campbell & Kelso 2021).

The initial course of management by personnel that can administer IV should follow these principles (Pumphrey 2003; Simons 2004; Simons et al. 1998, 2001):

1. Remove, where possible, the inciting agent.
2. Call for help.
3. Intramuscular (IM) injection of epinephrine at the earliest opportunity, followed by epinephrine by IM or IV injection as needed.
4. Placement of the patient in the supine position with the lower extremities elevated, unless there is prominent upper airway swelling which will prompt the patient to remain upright (and often leaning forward). If the patient is vomiting, placement of the patient semi-recumbent position with lower extremities elevated.
5. Ensure that supplemental oxygen is utilised.
6. Volume russification with IV fluids and if the patient is normotensive, isotonic (0.9%) saline should be infused at a rate of 125 mL/h to maintain venous access. In normotensive children, isotonic saline should be infused at an appropriate maintenance rate for weight to maintain venous access (only if personnel that can administer IV fluids is available).

**FIGURE 2:**
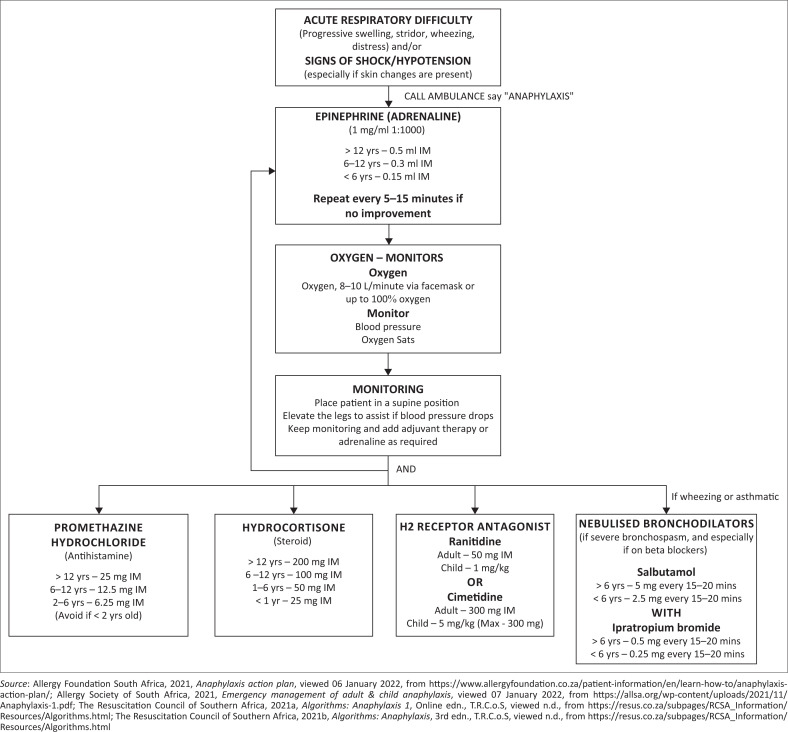
Anaphylaxis algorithm.

In a pharmacy setting the following should be adhered to regarding the management of anaphylaxis (Allergy Foundation South Africa 2021; Campbell & Kelso 2021; Pumphrey 2003; Simons et al. 1998, 2001; Simons 2004). An adapted algorithm for anaphylaxis management by pharmacists is given (Figure 2):

1. Remove the inciting agent.
2. Call for help – CALL AMBULANCE say ‘ANAPHYLAXIS’.
3. Assess airway, breathing and circulation.
4. Epinephrine (adrenaline) IM should be administered in the mid-outer aspect of the thigh (antero-lateral aspect of the middle third of the thigh).
5. Placement of the patient in the supine position with the lower extremities elevated, unless there is prominent upper airway swelling which will prompt the patient to remain upright (and often leaning forward). If the patient is vomiting, placement of the patient semi-recumbent position with lower extremities elevated. Place pregnant patients on their left side.
6. Supplemental oxygen (15 L/min flow rate or commercial high flow oxygen masks [providing at least 70% and up to 100% oxygen] should be administered).
7. Monitoring blood pressure, heart rate, and respiratory rate, as well as monitoring of oxygen saturation by pulse oximetry, is required for the duration of the episode.

#### Pregnant women

For a pregnant woman experiencing anaphylaxis, place the patient on her left side while providing a high flow rate of oxygen. Monitor her blood pressure, and if possible, perform foetal monitoring (Campbell & Kelso 2021).

### Adverse event following immunisation reporting

Theoretically, all AEFIs, including those that resolve spontaneously or are of minor clinical significance and those that are unexpected, undocumented, serious and severe should be reported (World Health Organization 2022). The South African Health Products Regulatory Authority (SAHPRA) recommends the use of the Med Safety Mobile App, the EML Clinical Guide Mobile App or the eReporting link to VigiFlow^®^ as mechanisms to report AEFI’s. Step-by-step assistance is available on the SAHPRA (2021) website.

## Implications and recommendations

This review serves to offer guidance for the management of allergy and anaphylaxis in a pharmacy setting. Immunisation in a pharmacy setting is increasing and it is likely that pharmacists will encounter patients with anaphylaxis. Future research needs to focus on re-assessing the scope, training and education of a pharmacist to include venipuncture and simulation-based training in the management of anaphylaxis for pharmacists and pharmacy students. Based on the findings of this review, it is recommended that pharmacists prioritise contacting an ambulance once it is identified that the patient is experiencing anaphylaxis. Focus needs to be kept on symptomatically managing the anaphylaxis until the ambulance arrives. As a result of IV administration not being within the scope of practice of a pharmacist, it is recommended that the patients’ legs be elevated to assist with the drop in blood pressure.

## Conclusion

The guidance provided should assist pharmacy personnel to manage allergy and anaphylaxis within the relevant scope of practice. The contents of the emergency tray should be regularly inspected, and personnel should be aware of the prompt intervention required to manage allergy and anaphylaxis within the scope of practice in order to reduce the risk of death.
